# Most Published Research Findings Are False—But a Little Replication Goes a Long Way

**DOI:** 10.1371/journal.pmed.0040028

**Published:** 2007-02-27

**Authors:** Ramal Moonesinghe, Muin J Khoury, A. Cecile J. W Janssens

## Abstract

While the authors agree with John Ioannidis that "most research findings are false," here they show that replication of research findings enhances the positive predictive value of research findings being true.

We know there is a lot of lack of replication in research findings, most notably in the field of genetic associations [1–3]. For example, a survey of 600 positive associations between gene variants and common diseases showed that out of 166 reported associations studied three or more times, only six were replicated consistently [4]. Lack of replication results from a number of factors such as publication bias, selection bias, Type I errors, population stratification (the mixture of individuals from heterogeneous genetic backgrounds), and lack of statistical power [5].

In a recent article in *PLoS Medicine*, John Ioannidis quantified the theoretical basis for lack of replication by deriving the positive predictive value (PPV) of the truth of a research finding on the basis of a combination of factors. He showed elegantly that most claimed research findings are false [6]. One of his findings was that the more scientific teams involved in studying the subject, the less likely the research findings from individual studies are to be true. The rapid early succession of contradictory conclusions is called the “Proteus phenomenon” [7]. For several independent studies of equal power, Ioannidis showed that the probability of a research finding being true when one or more studies find statistically significant results declines with increasing number of studies.

As part of the scientific enterprise, we know that replication—the performance of another study statistically confirming the same hypothesis—is the cornerstone of science and replication of findings is very important before any causal inference can be drawn. While the importance of replication is also acknowledged by Ioannidis, he does not show how PPVs of research findings increase when more studies have statistically significant results. In this essay, we demonstrate the value of replication by extending Ioannidis' analyses to calculation of the PPV when multiple studies show statistically significant results.

The probability that a study yields a statistically significant result depends on the nature of the underlying relationship. The probability is 1 - ß (one minus the Type II error rate) if the relationship is true, and a (Type I error rate) when the relationship is false, i.e., there is no relationship. Similarly, the probability that *r* out of *n* studies yield statistically significant results also depends on whether the underlying relationship is true or not. Let B*(p,r,n)* denote the probability of obtaining at least *r* statistically significant results out of *n* independent and identical studies, with *p* being the probability of a statistically significant result. B*(p,r,n)* is calculated as

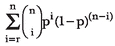
In this formula, *p* is 1 - ß when the underlying relationship is true and a when it is false. Let *R* be the pre-study odds and *c* be the number of relationships being probed in the field. The pre-study probability of a relationship being true is given by *R*/(*R* + 1). The expected values of the 2 × 2 table are given in Table 1. When *r* is equal to one, entries in Table 1 are identical to those in Table 3 of Ioannidis [6]. The probability that, in the absence of bias, at least *r* out of *n* independent studies find statistically significant results is given by (RB(1 - ß,*r*,*n*) + B(α,*r*,*n*))/(*R* + 1) and the PPV when at least *r* studies are statistically significant is RB(1 - ß,*r*,*n*)/((RB(1 - ß,*r*,*n*) + B(α,*r*,*n*)).

**Table 1:**
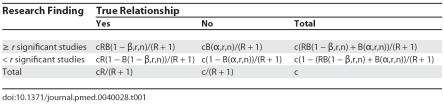
Research Findings and True Relationships in the Presence of Multiple Studies

## Positive Predictive Value as a Function of Study Replication

**Figure 1:**
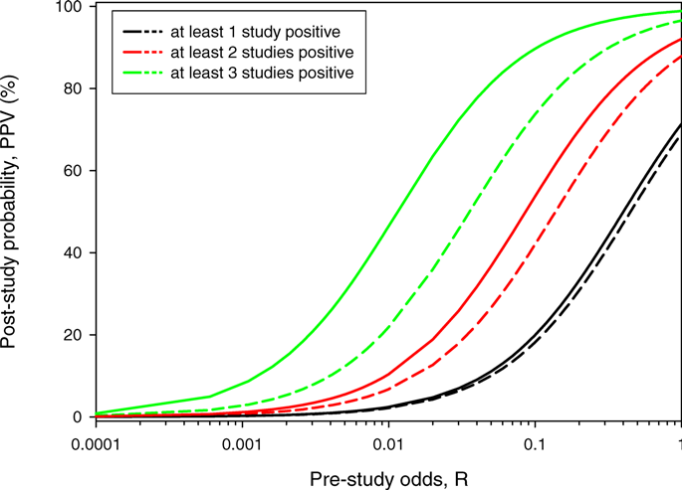
Probability of a True Relationship When At Least One, Two, or Three (Out of Ten) Studies Have Statistically Significant Results as a Function of the Pre-Study Odds of a True Relationship (α = 0.05) Dashed lines refer to power of 0.2 and solid lines to power of 0.8.

**Figure 2:**
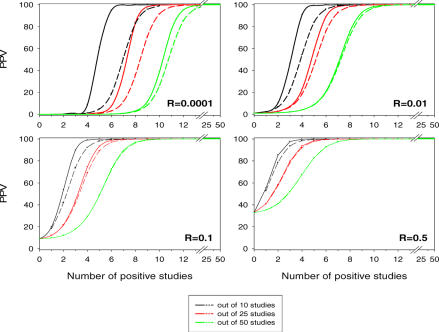
Positive Predictive Value for Research Findings Being True for At Least *r* Positive Studies Out of Ten, 25, and 50 Studies for Pre-Study Odds *R* of 0.0001, 0.01, 0.1, and 0.5 (α = 0.05) Dashed lines refer to power of 0.2 and solid lines to power of 0.8.

We examine the PPV as a function of the number of statistically significant findings. Figure 1 shows the PPV of at least one, two, or three statistically significant research findings out of ten independent studies as a function of the pre-study odds of a true relationship (*R*) for powers of 20% and 80%. The lower lines correspond to Ioannidis' finding and indicate the probability of a true association when at least one out of ten studies shows a statistically significant result. As can be seen, the PPV is substantially higher when more research findings are statistically significant. Thus, a few positive replications can considerably enhance our confidence that the research findings reflect a true relationship. When *R* ranged from 0.0001 to 0.01, a higher number of positive studies is required to attain a reasonable PPV. The difference in PPV for power of 80% and power of 20% when at least three studies are positive is higher than when at least one study is positive. Figure 2 gives the PPV for increasing number of positive studies out of ten, 25, and 50 studies for pre-study odds of 0.0001, 0.01, 0.1, and 0.5 for powers of 20% and 80%. When there is at least one positive study (*r* = 1) and power equal to 80%, as indicated in Ioannidis' paper, PPV declined approximately 50% for 50 studies compared to ten studies for *R* values between 0.0001 and 0.1. However, PPV increases with increasing number of positive studies and the percentage of positive studies required to achieve a given PPV declines with increasing number of studies. The number of positive studies required to achieve a PPV of at least 70% increased from eight for ten studies to 12 for 50 studies when pre-study odds equaled 0.0001, from five for ten studies to eight for 50 studies when pre-study odds equaled 0.01, from three for ten studies to six for 50 studies when pre-study odds equaled 0.1, and from two for ten studies to five for 50 studies when pre-study odds equaled 0.5. The difference in PPV for powers of 80% and 20% declines with increasing number of studies.

## Probability Distribution of Statistically Significant Results

Although the PPV increases with increasing statistically significant results, the probability of obtaining at least *r* significant results declines with increasing *r*. This probability and the corresponding PPV for pre-study odds of 0.0001, 0.01, 0.1, and 0.5 are given for ten studies in Table 2. When power is 20% and pre-study odds are 0.0001, the probability of obtaining at least three statistically significant results is 1% and the corresponding PPV is 0.3%. This probability and the corresponding PPV increase with increasing pre-study odds. For example, when *R* = 0.1, the probability of obtaining at least three significant results is 4% and the PPV is 74%. As expected, both the probability of obtaining statistically significant results and the corresponding PPV increase with increasing power. However, for very small *R* values (around 0.0001), the increase in power has a minimal impact in the probability of obtaining at least one, two, or three statistically significant results. When power is 80%, the probability of obtaining at least three statistically significant results is 1.2% and the corresponding PPV is 0.9% for *R* = 0.0001, and when pre-study odds are 0.1, the probability of obtaining at least three statistically significant results increases to 10% and the corresponding PPV to 90%.

**Table 2:**
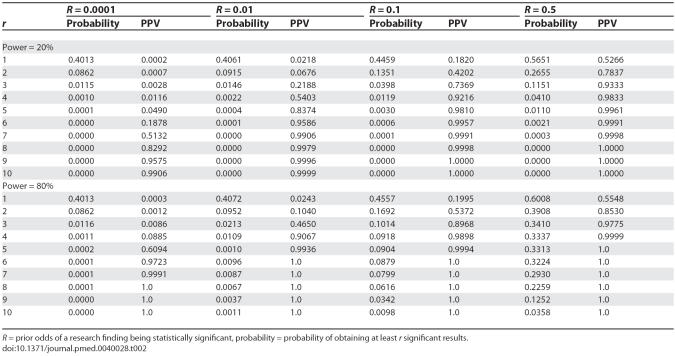
Probability of Obtaining At Least *r* Significant Results Out of Ten Studies when Pre-Study Odds Equal 0.0001, 0.01, 0.1, and 0.5

## Comment

The importance of research replication was discussed in a *Nature Genetics* editorial in 1999 lamenting the nonreplication of association studies [8]. The editor emphasized that when authors submit manuscripts reporting genetic associations, the study should include an effect size and it should contain either a replication in an independent sample or physiologically meaningful data supporting a functional role of the polymorphism in question. While we acknowledge that our assumptions of identical design, power, and level of significance reflect a somewhat simplified scenario of replication, we quantified the positive predictive value of true research findings for increasing numbers of significant results. True replication, however, requires a precise process where the exact same finding is reexamined in the same way. More often than not, genuine replication is not done, and what we end up with in the literature is corroboration or indirect supporting evidence. While this may be acceptable to some extent in any scientific enterprise, the distance from this to data dredging, moving the goal post, and other selective reporting biases is often very small and can contribute to “pseudo” replication.

Replication does not mean that we can have underpowered studies; even when we have several underpowered studies replicate a finding, the PPV remains low. Good replication practices require adequately powered studies. More generally, meta-analysis is a more useful approach to assess the totality of evidence in a body of work. Ioannidis discussed the importance of meta-analysis, and its weaknesses in cases where even the meta-analysis is underpowered.

Our calculations have not considered the possibility of bias, i.e., selective reporting problems that may change some “negative” results to “positive” or may leave “negative” results unpublished. John Ioannidis has shown that modest bias can decrease the PPV steeply [6]. Therefore if replication is to work in genuinely increasing the PPV of research claims, it should be coupled with full transparency and non-selective reporting of research results. Note that when hypotheses are one-sided, according to our definition of replication, we only consider hypotheses that are in the same direction. Under this definition, statistically significant results in both directions do not arise. However, in meta-analysis, one can combine results that are significant in opposite directions. Calculations in a formal meta-analysis may not square fully with the inference presented here, since meta-analysis would incorporate both effect sizes and their uncertainty rather than just the “positive” versus “negative” inference. For example, we may have the necessary number of “positive” studies, but if the observed “positive” effects are small and all the other studies have trends in the opposite direction, the summary effect may well be null.

In summary, while we agree with Ioannidis that most research findings are false, we clearly demonstrate that replication of research findings enhances the positive predictive value of research findings being true. While this is not unexpected, it should be encouraging news to researchers in their never-ending pursuit of scientific hypothesis generation and testing. Nevertheless, more methodologic work is needed to assess and interpret cumulative evidence of research findings and their biological plausibility. This is especially urgent in the exploding field of genetic associations.

## Abbreviations

PPV: positive predictive value
